# EFFECTS OF A STRENGTHS-BASED DYADIC PROGRAM ON THE HEALTH OF PERSONS WITH MILD COGNITIVE IMPAIRMENT AND THEIR FAMILY

**DOI:** 10.1093/geroni/igz038.420

**Published:** 2019-11-08

**Authors:** Doris Yu, Sheung-Tak Cheng, Polly Li, Fan Zhang

**Affiliations:** 1 The Chinese University of Hong Kong, Hong Kong, Hong Kong; 2 The Education University of Hong Kong, Hong Kong, Hong Kong

## Abstract

Mild cognitive impairment (MCI) is characterized by cognitive impairment cognition, neuro-psychiatric symptoms particularly depressed mood, and compromised self-image. This RCT evaluated the effects of a strength-based dyadic program, which supported MCI care dyads for effective symptom and disease management and active social engagement, on the dyadic health outcomes. From Dec 2017-Jan 2019, 103 care dyads were recruited in an elderly community center. By adopting the strength-based and person-centered approach, this 14-week program worked out a dyadic biographies for care dyads to increase identification and recognition of their own strengths. Then, six interactive educative-counseling sessions focusing on cognitive and behavioral symptom management, social interaction and gauge capability were delivered, with four sessions for the MCI clients in small groups and two sessions for the care dyads in their home environment. The control group received usual care. Dyadic health outcomes were measured at baseline, upon program completion and three months thereafter. The results of Generalized Estimating Equations showed that the strength-based dyadic program significantly improved the objective memory (post-test: β=-0.179, p=0.001), and subjective memory (post-test: β=7.542. p=0.007; 3-month: β=7.842. p=0.012), neuro-psychiatric symptoms (post-test: β=2.822, p=0.001; 3-month: β=3.038. p=0.007), and depression (post-test: β=2.665, p=0.017; 3-month: β=3.556. p=0.007). It also improved the caregiver stress in symptom management at post-test (β=2.822. p=0.001) and depression at the 3-month endpoint (β=1.510. p=0.015). Identifying and mobilizing the strengths of the clients with MCI and their family caregivers not only promote a more assertive coping experience and positive self-image for the care dyads, but also improve their health outcomes.

